# A Selenium Containing Inhibitor for the Treatment of Hepatocellular Cancer

**DOI:** 10.3390/ph9020018

**Published:** 2016-03-24

**Authors:** Hephzibah Rani S. Tagaram, Dhimant Desai, Guangfu Li, Dai Liu, C. Bart Rountree, Kavitha Gowda, Arthur Berg, Shantu Amin, Kevin F. Staveley-O’Carroll, Eric T. Kimchi

**Affiliations:** 1 Department of Surgery, Pennsylvania State University, Hershey, PA 17033, USA; htagaram@gmail.com (H.R.S.T.); kxs58@psu.edu (K.G.); 2 Department of Pharmacology, Pennsylvania State University, Hershey, PA 17033, USA; 3 Medical University of South Carolina, Charleston, SC 29425, USA; liggu@musc.edu (G.L.); dliu1@hmc.psu.edu (D.L.); ocarrollk@health.missouri.edu (K.F.S.-O.C.); kimchie@health.missouri.edu (E.T.K.); 4 Bon Secours Pediatric Associates, 5875 Bremo Road, Richmond, VA 23226, USA; crountree@hmc.psu.edu; 5 Department of Public Health Sciences, Pennsylvania State University, Hershey, PA 17033, USA; aberg1@hmc.psu.edu

**Keywords:** inhibition, hepatocellular cancer, selenium, apoptotic and angiogenesis markers

## Abstract

Hepatocellular carcinoma (HCC) is the third most deadly cancer in the world. New treatment strategies are desperately needed due to limited standard therapies. Activation of the Erk, Akt, and STAT3pathways is implicated in the prognosis of HCC. The *Se*,*Se′*-1,4-phenylenebis(1,2-ethanediyl) bisisoselenourea (PBISe), is a selenium-containing MAPK and PI3 kinase inhibitor, effectively inhibit tumorigenesis in a variety of experimental models. The aim of our study is to demonstrate the potential role of PBISe in the treatment of HCC. The anti-proliferative and pro-apoptotic ability of PBISe is studied *in vitro* in four human HCC cell lines and *in vivo* in a spontaneous murine HCC model. Inhibition of cancer growth was performed by cell viability assay and apoptosis by caspase 3/7, PARP cleavage, annexin-V, and TUNEL assays. Role of PBISe on PI3 kinase, MAPK and STAT3 signaling is determined by Western blotting. *In vivo* effects of PBISe on tumor sizes were monitored using MRI in a spontaneous murine HCC. Liver tissues from the PBISe-treated mice are analyzed for angiogenesis, proliferation, and signaling pathway markers. Overall, PBISe activated caspase-3/7 and increased DNA fragmentation, which is positively correlated with the increased PARP cleavage. PBISe promoted apoptosis by inhibiting PI3K, MAPK, and STAT3 signaling with significant reduction in the tumor sizes (*p* < 0.007). PBISe-treated tumors reduced survival marker PCNA, and angiogenesis markers Vegf-A, Vegf-R3 and CD34. These results demonstrate the chemotherapeutic effects of PBISe, by inhibiting tumor growth and facilitating tumor apoptosis for HCC treatment.

## 1. Introduction

Human hepatocellular cancer (HCC) is one of the most lethal cancers in the world [1,2]. The median survival following diagnosis is approximately six to 20 months. Surgical tumor resection or liver transplantation are the best therapies for improving survival in patients with HCC. However, only a minority of patients are candidates for these procedures, because of tumor extent or underlying liver dysfunction. Sorafenib is the only reference standard systemic treatment for advanced HCC with a modest survival benefit [3,4]. New alternative treatment strategies are needed for HCC. Both oxidative stress and inflammatory mechanisms have been implicated in the pathophysiology of HCC [5]. The use of dietary antioxidants and micronutrients, such as selenium, has been proposed as an effective means for successful management of human HCC. Trace elements, such as vanadium and selenium, are involved in several major metabolic pathways, as well as antioxidant defense systems [6,7]. Selenium is known to be an effective chemopreventive agent in chronic diseases and various cancers [8,9,10,11,12,13,14,15,16,17]. A new selenium compound with less toxicity might benefit HCC patients who have significant liver dysfunction [18].

A selenium compound, called *S*,*S*’-1,4-phenylenebis (1,2-ethanediyl) bisisothiourea (PBIT), is a Akt/iNOS inhibitor, which effectively inhibits colonic aberrant crypt foci (ACF) and esophageal cancers at high doses in animal models [19,20]. However, to improve this drug’s restricted therapeutic efficacy and relative impotency, we have developed a novel selenium analog of PBIT by replacing sulfur with selenium, *i.e.*, *Se*,*Se*′-1,4-phenylenebis (1,2-ethanediyl) bisisoselenourea (PBISe). This newly developed novel selenium compound, PBISe, was found to be more potent than its isosteric sulfur analog, PBIT [21]. PBISe has shown effectiveness on various epithelial cancer cells, such as colon cancer [22], melanoma [23], and lung cancer [24]. PBISe works by down-regulating Akt signaling, decreasing cancer cell viability and promoting apoptosis. Topical application of PBISe has shown to retard melanocytic lesion development in laboratory generated skin by 70% to 80% and in animal skin by approximately 50% compared to those treated with PBIT or vehicle control [19]. In treated mice, body weights and serum biomarkers indicative of toxicity in the liver, heart, kidney, intestine, pancreas and adrenal gland showed negligible differences compared to untreated mice [19]. More importantly, our laboratory examination of biomarkers for major organ related toxicity in the blood of F344 rats was found to be normal at all concentrations tested (PBISe, 1 ppm to 20 ppm as Se) (*p* < 0.01, unpaired *t* test) when compared to control animals (unpublished results). Considering PBISe as a promising drug because of its negligible systemic toxicity and chemotherapeutic efficacy, we propose to investigate the *in vitro* and *in vivo* effects of PBISe and the mechanisms involved in a preclinical model of spontaneous liver cancer.

In the present study, the mechanism underlying the PBISe induced apoptosis in primarily cultured human HCC cells was explored. The Akt/PI3K signaling pathway plays a significant role in carcinogenesis and drug resistance in different types of cancer, including HCC, making these pathways a potential targets for cancer treatment. STAT3 is another transcriptional factor involved in immune responses, inflammation and tumorigenesis, and was found to be critical for compensatory liver regeneration and chemically induced HCC development.

Angiogenesis the growth of capillary vessels from existing ones is obligatory for growth, progression and metastasis of solid cancers including HCC [25]. Elevated Vegf [26,27] and Vegf-R3 [28] is expressed in several tumors, including HCC, to promote tumor development is associated with Akt activation [29,30]. Decrease of micro vessel density (MVD) using Selenium was demonstrated in mammary cancer. We intend to explore the role of angiogenesis by PBISe in the murine model of HCC. To define the potential contribution of angiogenesis, we have examined the expression of Vegf-A, Vegf-R3, and CD34.

The effect of PBISe on the Phosphotidylinositol 3-Kinase (PI3K), Mitogen-activated protein kinase (MAPK) and Signal transducer and activator of transcription 3 (STAT3) pathways were evaluated. We demonstrated that PBISe arrests cell growth of HCC by the caspase 3/7 induced apoptosis with increase in cleaved PARP, and decrease in the phosphorylation of MAPK, PI3K and STAT3 pathways. *In vivo* treatment of spontaneous tumors with PBISe demonstrates PBISe as a promising selenium compound for the treatment of established HCC.

## 2. Results

### 2.1. PBISe Inhibits Survival of Human HCC

**Figure 1:**
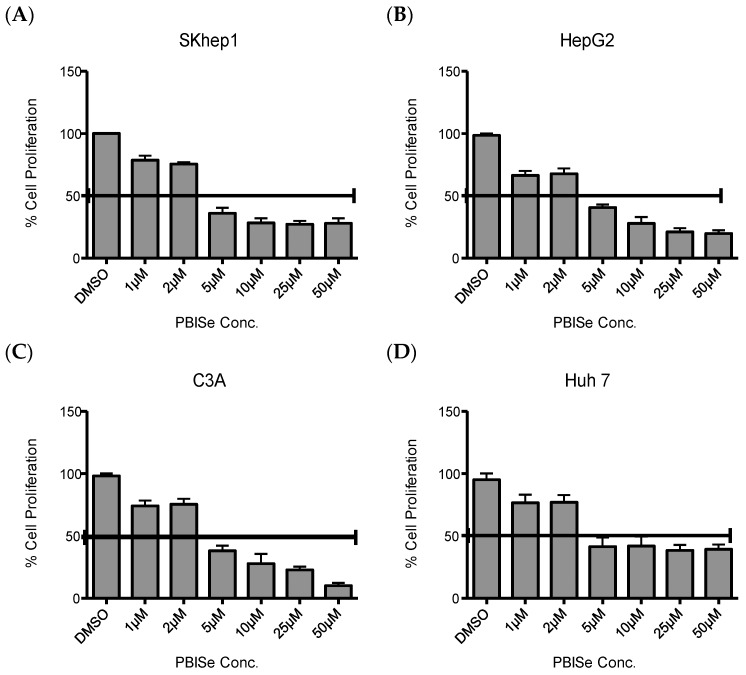
PBISe treatment inhibits the growth of HCC *in vitro.* Four different HCC cells lines were used to demonstrate the inhibition process. (**A**) Skhep1; (**B**) HepG2; (**C**) C3A; (**D**) Huh-7. PBISe inhibits proliferation of all HCC cell lines in a dose dependent manner. The HCC cells were seeded onto 96-well plates (5000 per well) in MEM with 10% FBS and allowed to attach overnight. Cells were then treated with PBISe or DMSO at indicated concentrations the following day, as shown in the figure. After 48 h of treatment, cell proliferation assays were performed using Cell’Titer 96 Aqueous Assay Kit (Promega) according to manufacturer’s instructions. Results of three individual experiments were plotted as percentage of cell proliferation. The bars indicate Mean ± S.E.

To determine the mechanism by which PBISe inhibited human liver cancer cells, rate of cellular proliferation with PBISe treatment was performed. For this work, we utilized four independent human HCC cell lines: Skhep1 cells, HepG2 cells, C3A cells, and Huh7 cells. Figure 1 demonstrates that PBISe inhibits the growth of all four human HCC lines in a dose dependent manner. The IC_50_ values for all the cell lines used ranged between 2 μM and 5 μM of selenium. Thus, PBISe inhibits the survival of invasive Skhep1 and non-invasive HepG2, C3A and Huh7 human HCC. The IC_50_ values of PBISe were 2.69, 2.69, 3.07, and 2.86 μM for SKhep1, HepG2, C3A, and Huh7, respectively.

### 2.2. PBISe Induces Apoptosis in Human HCC

**Figure 2:**
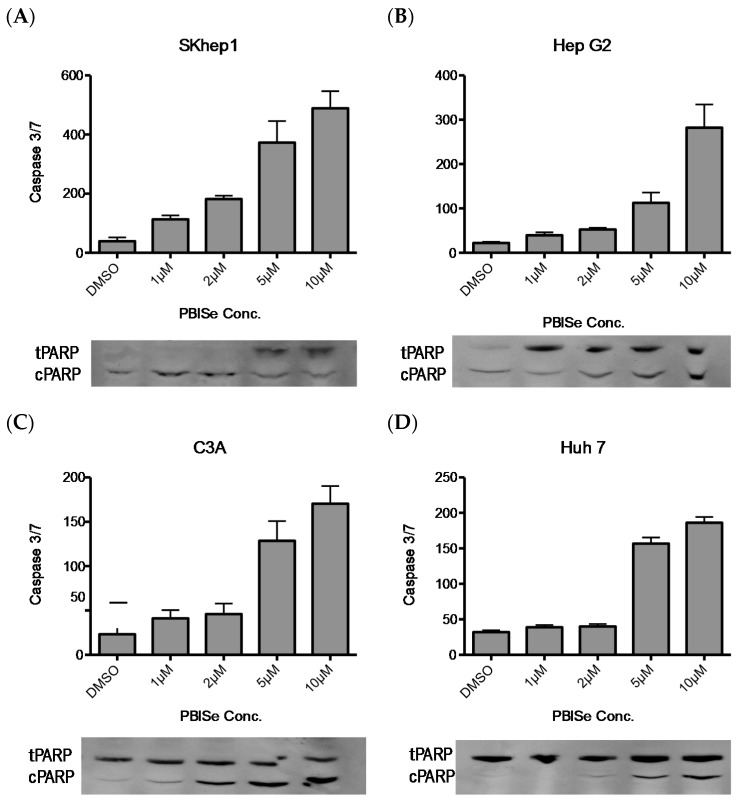
PBISe induces caspase dependent apoptosis of human HCC in a dose-dependent fashion. Four different cell lines were used to demonstrate the apoptotic process. (**A**) Skhep1; (**B**) HepG2; (**C**) C3A; (**D**) Huh-7. HCC cells were seeded onto 96-well plates (5 × 10^3^/well) in MEM with 10% FBS and allowed to attach overnight. Cells were then treated with PBISe or DMSO at indicated concentrations the following day, as shown in the figure. After 48 h of treatment, cell apoptosis assays were performed using Apo-one Homogeneous Caspase-3/7 Assay (Promega) according to the manufacturer’s instructions. n = 3; error bar represent Mean ± S.E. The protein expression of cleaved PARP analysis was performed by Western blot analysis as shown in figure for all cell lines.

It was observed that human HCC cell lines treated with PBISe resulted in the induction of caspase 3/7 in a dose dependent manner as shown in the bar graph (Figure 2). This caspase activation was also accompanied by the increase in cleavage of poly (ADP-ribose) polymerase (cPARP) with anti-cPARP antibody, performed using Western blotting (shown below), corresponding to the bar graph (Figure 2). These results demonstrate that PBISe is able to effectively activate apoptosis in all four HCC cell lines studied.

**Figure 3:**
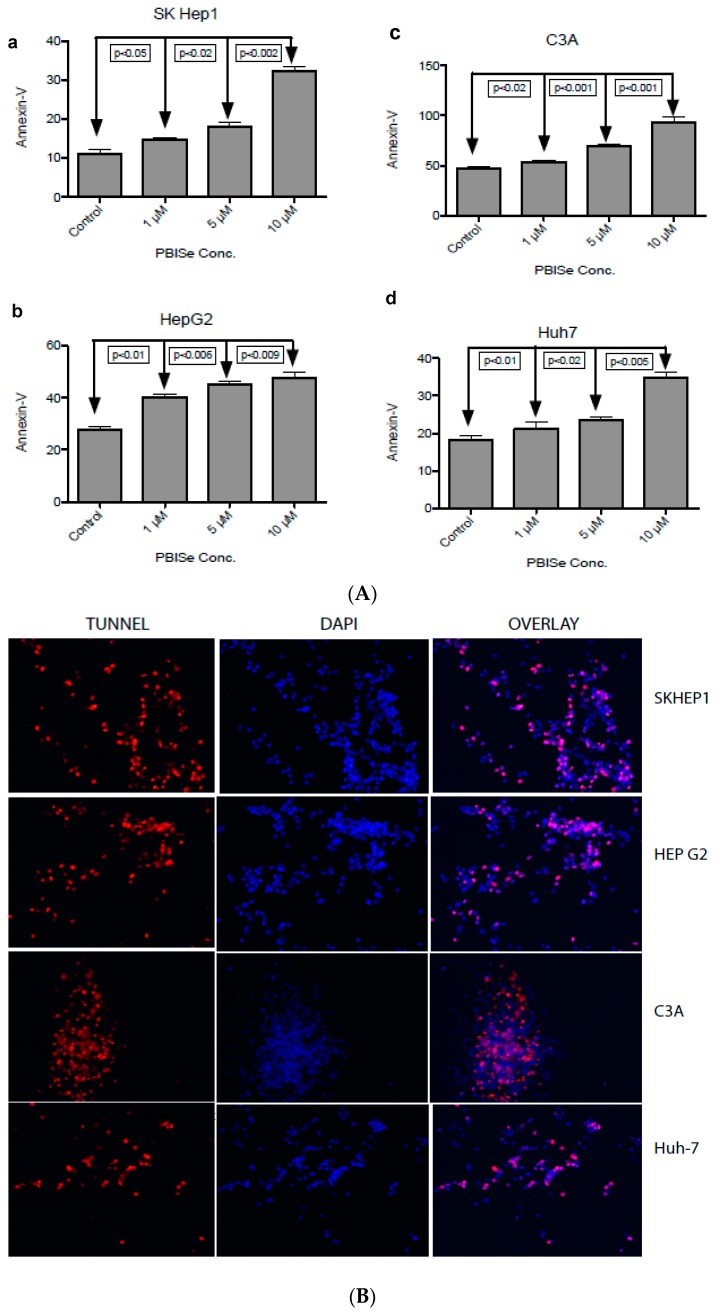
PBISe induces late stage apoptosis resulting in DNA fragmentation. Four different cell lines were used to demonstrate the late apoptotic process. (**a**) Skhep1; (**b**) HepG2; (**c**) C3A; (**d**) Huh-7. The cells were grown in 100 mm petridishes in 10% MEM, and then treated with different concentration of the drug for 48 h. Approximately 1 × 10^6^ treated cells were suspended in 1X binding buffer. One-hundred microliters of suspended cells containing 1 × 10^5^ cells were stained with 5 μL of FITC labeled annexin-V and 5 μL of Propidium iodide (PI) for 15 min at RT in dark. Apoptosis is measured by flow cytometry within one hour of staining. Graph showing the total apoptotic cell death from three quadrants representing the PI stained cells (early apoptotic), annexin-V staining (Late apoptotic) and double positive (early and late apoptotic). The p values are determined for all the indicated concentrations compared to control (**A**). As an indication of late stage apoptosis, PBISe induced DNA fragmentation of Skhep1, HepG2, C3A and Huh7 cell lines was performed by Tunnel assay. The cells are grown on the coverslip in a 6 well plate, treated with 10 μM conc. of PBISe for 48 h. The treated coverslips were fixed with paraformaldehyde followed by precooled ethanol and acetic acid. The coverslips were labeled with Apo tag red for Tunnel (red) and counterstained with DAPI nuclear stain (blue). The pictures were taken with Leica confocal microscopy (**B**).

PBISe induced apoptotic cell death was further confirmed with annexin-V staining of the HCC using flow cytometry. PBISe treatment demonstrated a dose dependent increase in annexin-V staining. Significance were calculated for 1 μM, 5 μM and 10 μM compared in all the four cell lines studied. The *p*-values for SKhep1 are *p* < 0.05, *p* < 0.02 and *p* < 0.002, respectively. The *p*-values for HepG2 are *p* < 0.01, *p* < 0.006, and *p* < 0.009 respectively. The *p*-values for C3A are *p* < 0.02, *p* < 0.001 and *p* < 0.001, respectively. The p-values for Huh-7 are *p* < 0.01, *p* < 0.02, *p* < 0.005 (Figure 3A). In order to demonstrate the effect of PBISe on late stage apoptosis, the treated HCC were stained for DNA degradation in apoptotic cells at 10 μM concentration of PBISe Terminal transferase dUTP Nick End Labeling (TUNEL) assay was used to determine this late stage of apoptosis (Figure 3B).

### 2.3. PBISe Inhibits MAPK, PI3K and STAT3 Pathways

**Figure 4:**
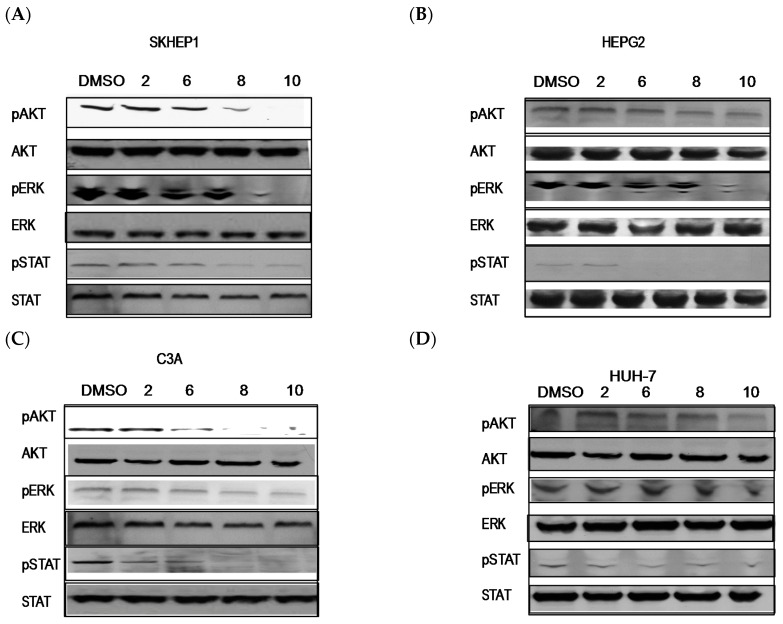
PBISe inhibits AKT/PI3K, MAPK/ERK and STAT3 signaling in human HCC. Four different cell lines were used to demonstrate the late apoptotic process. (**A**) Skhep1; (**B**) HepG2; (**C**) C3A; (**D**) Huh-7. 1 × 10^6^ cells were treated with increasing conc. of PBISe 2–10 μM for 48 h. Cell lysates were prepared and Western blot analyses were performed. As observed in the figure, pAKT, pERK and pSTAT3 were decreased in a dose dependent manner, suggesting that PBISe has a role not only on PI3K and MAPK but also on STAT3 signaling pathway.

Activation of ERK and AKT is implicated in the poor prognosis in HCC. To analyze the effect of PBISe treatment on the MAPK and PI3K signaling pathways, we treated the four HCC cell lines with different concentrations of PBISe (2–10 μM). PBISe treatment inhibited pAkt, pErk and pSTAT3 in all four cell lines in a dose dependent manner. This inhibition of oncogenic PI3K and MAPK signal pathways indicates a potential mechanism by which PBISe inhibits cell proliferation and induces apoptosis in HCC (Figure 4).

### 2.4. PBISe Inhibits In Vivo HCC Growth by Restricting Angiogenesis and Inducing Tumor Apoptosis

**Figure 5:**
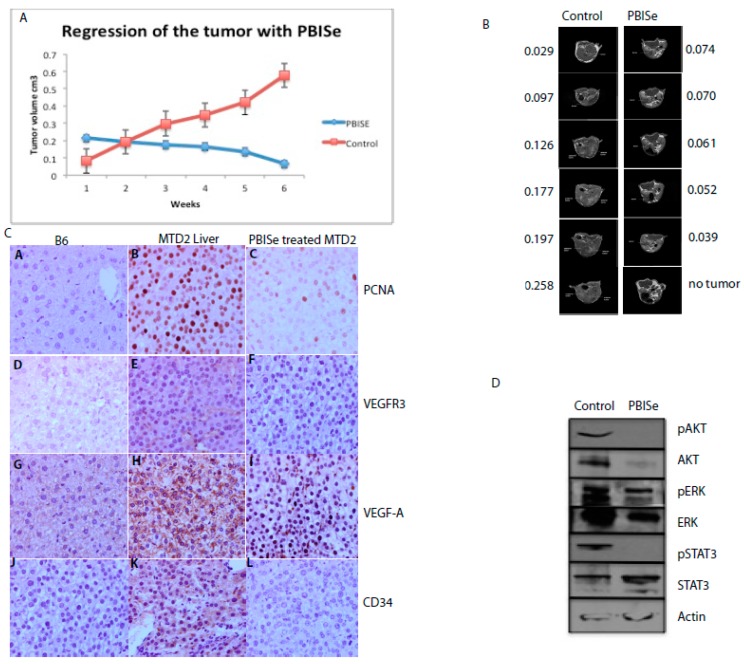
PBISe inhibits the growth of spontaneous murine HCC mediated by apoptosis and regulating angiogenesis. MRI was performed to confirm the initial size of the tumor prior to the start of the PBISe treatment. PBISe (2.5 ppm as se) was administered intra peritoneal (i.p.) to the HCC mice, every alternate day for about 6 weeks. (**A**) shows the regression of the tumor size (*n* = 6). MRI images taken at week 1 through week 6 are shown (**B**). Role of PBISe on angiogenesis is demonstrated on the PBISe treated murine HCC by IHC. PBISe decreased VEGFR3, VEGF and CD34 (**C**). Additionally, the inhibition of pAkt, pErk and pSTAT3 is demonstrated (**D**).

A potential anti angiogenic effect of selenium (Se) in the chemotherapy of murine HCC was demonstrated. The effect of PBISe on inhibiting HCC tumors was evaluated on SV40 T antigen transgenic mice. The mice spontaneously develop HCC, resulting from transgenic expression of SV40 T-ag under urinary promoter. The mice were given 5 ppm (as selenium) tolerance dose of PBISe intra peritoneal (i.p.) injections every alternate day for six weeks. The mean tolerance dose (MTD) of selenium was determined from previous experiments in Min (Multiple intestinal neoplasias) mice. MRI was performed to monitor the tumor sizes before and during treatment. The tumor sizes at the beginning of experiments were between 100 and 150 mm^3^. After six weeks of treatment with PBISe the sizes were reduced to 50 mm^3^ to no tumor whereas, the tumor which are in the control group, were increased to 270 mm^3^. The results indicate the ability of PBISe to regress the HCC tumors *in vivo* (Figure 5) (*p* < 0.007). A regression-based statistical analysis was performed to quantify the effects of treatment, time, and their interaction on tumor size. A significant interaction (*p* < 0.001) between treatment is present suggesting tumor growth in controls is much different than tumor growth in treatment group (Figure 5A). MRI for one of the mice from control group and one from treated group is shown (Figure 5B). Previous studies have demonstrated no signs of systemic toxicity in the animals, suggesting its safe use for treatment of various types of cancers [21]. The harvested tumors from PBISe treated murine HCC demonstrated decrease in the angiogenesis markers like Vegf-A, Vegf-R3 and CD34 along with the proliferation marker PCNA compared to the control (Figure 5C). Decrease in phosphorylation of Akt, Erk and STAT3 were demonstrated (Figure 5D).

## 3. Discussion

Hepatocellular Cancer (HCC) is one of the most common and deadliest cancers in the world and its incidence has been constantly rising in the United States. Although sorafenib has become the treatment of choice for advanced HCC, there are still unsettled issues regarding the optimal use of sorafenib [4]. The treatment of HCC is quite challenging and there is a need for new options to explore possible alternative chemopreventive and chemotherapeutic strategies. Both oxidative stress and inflammatory mechanisms have been implicated in the pathophysiology of HCC. The use of dietary antioxidants and micronutrients like selenium has been proposed as an effective means for successful management of human HCC [31]. Selenium has shown to be involved in the prevention of numerous chronic illnesses such as several specific cancers [32] including liver cancer [33]. Several investigators have reviewed the potential inverse correlation ship between selenium levels and the occurrence of HCC [8,29,30]. It is recognized as being an essential component of a number of enzymes in which it is present as the amino acid selenocysteine (SeCys). Selenium compounds have also been found to inhibit tumorigenesis in a variety of animal models and recent studies indicate that supplemental Se in human diets may reduce cancer risk [27]. Selenium also provided evidence for the chemopreventive effects on HCC [33].

PBISe is a selenium compound found to be chemopreventive and chemotherapeutic in melanoma [23] and lung cancer [24]. Our study was intended to demonstrate the effect of PBISe on human HCC to further our understanding of the functional role of PBISe on tumorigenic signaling pathways.

Dysregulated signaling through the MEK/ERK and PI3K/Akt pathways is often the result of genetic alterations in critical components in these pathways or upstream activators [34]. Unrestricted cellular proliferation and decreased sensitivity to apoptotic-inducing agents are typically associated with activation of these pro-survival pathways [35]. Activation of AKT and ERK signaling are implicated in the poor prognosis of HCC and ERK activation in the cancer tissue is associated with hepatitis C virus infection. Activation of ERK1/2 in HCC indicates aggressive tumor behavior and constitutes an independent prognostic factor [36]. The Akt /PI3K signaling pathway also plays a significant role in carcinogenesis and drug resistance in different types of cancers including HCC, making Akt a potential target for cancer treatment [36,37]. Akt pathway has been identified as a significant risk factor for early disease recurrence and poor prognosis in HCC patients. Tumors with activated PI3K/Akt signaling have been shown to become more aggressive and promote HCC growth [38]. Moreover, cell survival is usually controlled by the combination effects of different MAPK signal pathways rather than by any single one. The activated pathway also makes the cancer more aggressive and more likely to spread to other organs. Our results on PI3 kinase pathway, demonstrated in human HCC offers a great promise for more effective therapies for the HCC.

Extracellular signal regulated mitogen activated protein kinase signaling is a critical growth regulatory pathway for HCC. It is clearly evident that PBISe is capable of many signaling pathways involved in survival and apoptosis of liver, three of the mitogen-activated protein kinase (MAPK) signaling pathways, extracellular-regulated kinase 1/2 (ERK1/2) have been widely studied. ERK pathway is important in differentiation and proliferation, as well as in cell survival of HCC [39]. Effect of selenium treatment and Selenoprotein M overexpression on the MAPK pathway to attenuate α/γ-secretase-mediated proteolysis and Tau phosphorylation to protect brain function [40]. However ERK expression in HCC is associated with increased tumor size, histologic progression and intrahepatic metastasis. We observed a decrease in the phosphorylated ERK with selenium in HCC cells. It could be possible that the balance and integration between the signals may widely vary in different tumors, like that of HCC, that are important for the outcome and the sensitivity to drug therapy.

Accumulating evidences indicate that chemotherapeutic agents induce tumor regression through inhibition of proliferation and/or activation of apoptosis. Apoptosis has been identified as a critical mechanism for cancer chemoprevention by Se compounds. Changes that happened during apoptosis, including cell shrinkage, formation of apoptotic bodies, accumulation of sub-G1 cell population, DNA fragmentation and nuclear condensation were observed in cancer cells treated with various concentrations of PBISe [23]. In mammalian models, two major apoptotic pathways have been identified, including the death receptor-mediated (extrinsic) pathway and the mitochondrial apoptotic (intrinsic) pathway. Both pathways involve the proteolysis of various cellular components initiated by activated caspases, a family of cysteine proteases. Our study shows that PBISe induced apoptotic cell death was accompanied by marked activation of caspase-3 and caspase-7. Selenium has proapoptotic [41] and antiangiogenic [29] properties that may be important for cancer chemoprevention. The PARP is a substrate of the caspase-3 protease during apoptosis; therefore, we examined cell lysates for PARP cleavage by western blot analysis, demonstrating the induction of cleaved PARP and its role in the induction of apoptosis. PBISe has demonstrated an increasing body of evidence, showing caspase dependent apoptosis is an important process for the regulation of pathogenesis of liver. 

PBISe was demonstrated to prevent and also treat melanoma development in the skin [23]. We demonstrated similar effects on HCC murine mice. PBISe significantly increase the survival of HCC murine mice by approximately five-fold. We have preliminary results demonstrating the mean tolerance dose (MTD) of PBISe as 5–10 ppm (as selenium) from Min mice. Several studies have focused on the effects of selenium on the *in vivo* models of hepatocarcinogenesis. Xu and coworkers have shown that green tea extracts enriched with selenium suppressed HCC in mice, which were xenografted with human hepatoma HepG2 [42]. Selenium significantly decreased the spontaneous liver tumorigenesis in CBA mice [43]. Novoselov and coworkers [44] showed that selenium supplementation in the diet-inhibited hepatocarcinogenesis and decreased cell proliferation in Myc transgenic mice, which are characterized by disrupted redox homeostasis and develop liver cancer by six months of age [45]. This is the first report, demonstrating the survival of HCC murine mice with PBISe, a novel selenium compound.

Selenium has been shown to inhibit angiogenesis in 1-methyl-1-nitroso urea (MNU)-induced rat mammary tumors through the suppression of Vegf expression and inhibition of gelatinolytic activity of MMP-2. The expression of Vegf-R3 is up regulated in >75% of HBxAg positive hepatocellular carcinoma (HCC) nodules. Vegf-R3 up-regulation correlates with the expression of HBxAg, is associated with decreased survival in tumor bearing patients. Evidence of selenium compounds such as dimethyl selenone, diphenyl selenone, sodium selenite, or Se methyl selenocysteine in reversal of pro angiogenesis effect of arsenic was demonstrated and mediated by ERK1/2 signal transduction pathway [46].

## 4. Experimental Section

### 4.1. Synthesis of PBISe

PBIT was synthesized as previously described [19,20]. PBISe, an isosteric selenium analog of known iNOS inhibitor PBIT, was prepared in good yield as reported [21]. In general, the methyl ester of 1,4-phenylenediacetic acid on reduction with LiAlH_4_ in THF yielded dihydrodiol in good yield. The diol was converted to the corresponding dibromo derivative by reacting with carbon tetrabromide and triphenyl phosphine [19]. The Dibromo derivative was reacted with thiourea or selenourea to yield the desired product dihydrobromide salt of PBIT and PBISe, respectively. Structures of PBIT and PBISe were confirmed by proton Nuclear Magnetic Resonance (NMR) (BRUKER, Billerica, MA, USA) and by Mass Spectrometry (MS) (ABSciex 5600 TripleTOF MS, Framingham, MA, USA). Purity of the compounds was confirmed by reverse phase High Performance Liquid Chromatography (HPLC) (Waters Corporation, Milford, MA, USA).

### 4.2. Cell Lines and Culture Conditions

The human hepatocellular carcinoma cell lines Skhep1, HepG2, C3A and Huh7 were included in the study. The cell culture base medium was an Invitrogen-formulated Dulbecco Minimum Essential Medium (DMEM) with the following components: 10% heat-treated fetal bovine serum at 56 °C for 1 h (FBS; Hyclone); Pen-Strep 100X Solution (Invitrogen, Carlsbad, CA, USA); Kanamycin 100X (Invitrogen) and Sodium bicarbonate. C3A and Huh7 cells were maintained in DMEM with additional Non-Essential Amino Acids (Invitrogen).

### 4.3. Cell Viability and Proliferation Assay

The viability of HCC cells following treatment with PBISe, was measured using the 3-(4, 5-dimethylthiazol-2-yl)-5-(3-carboxymethoxyphenyl)-2-(4-sulfophenyl)-2H-tetrazolium (MTS) assay (Promega, Madison, WI, USA). A total of 5000 cells/well were cultured in 100 μL of DMEM-10% FBS in a 96 well plate for 24 h respectively for each HCC cell line. These were treated with either PBS vehicle or increasing concentrations (2, 4, 6, 8, 10 μmol/L) of PBISe for 48 h. Cellular viability was measured using the Cell’Titer 96 Aqueous Assay Kit using MTS reagent (Promega). IC values (μmol/L) for each compound in respective cell lines was determined from three independent experiments using GraphPad Prism version 4.01 (GraphPad, San Diego, CA, USA).

### 4.4. Apoptosis

HCC cells were plated at 5 × 10^3^ cells per well in 96-well tissue culture plates and grown in 10% serum fortified media for 48 h prior to treatment. Cells were exposed to DMSO, or PBISe for 48 h in media containing 1% FBS. Caspase 3/7 enzymatic activity was measured following treatment using an Apo-ONE Homogeneous Caspase 3/7 Assay according to the manufacturer’s instructions (Promega). Caspase 3/7 activity was determined by measuring the fluorescence of the cleaved substrate using a micro plate reader (excitation 498 nm; emission 521 nm).4.5. Annexin V Assay

HCC cells were plated at 1 × 10^5^ cells per well in 6-well tissue culture plates and grown in 10% serum media for 48 h prior to treatment. Cells were exposed to DMSO, PBISe for 48 h in media with FBS. Cells were harvested and stained with PE-labeled annexin V (EMD Biosciences, San Diego, CA, USA) and 7-aminoactinomycin D (Invitrogen, Carlsbad, CA, USA) and analyzed by flow cytometry.

### 4.6. Terminal Deoxynucleotide Transferase dUTP Nick End Labeling (TUNEL) Assay

HCC cells were plated at 1 × 10^4^ cells per well in 8-well chamber slides and grown in 10% serum fortified media for 48 h prior to treatment. Cells were exposed to DMSO, PBISe for 48 h in media containing 1% FBS. Fragmented DNA of apoptotic cells was stained using an ApopTag Red *in Situ* Apoptosis Detection Kit according to the manufacturer’s instructions (Chemicon, Temecula, CA, USA) and visualized by fluorescence microscopy using appropriate filters.

### 4.7. Western Blot Analysis

Cell lysates were prepared in lysis buffer (0.1% NP40, 50 mM HEPES, 137 mM NaCl, 10 mM Na4P2O7, 50 mM NaF, 5 mM β-glycerolphosphate, 1 mM EGTA, 2 mM EDTA, 1% glycerol, containing protease inhibitor (EMD Biosciences, Billerica, MA, USA) for 20 min on ice followed by centrifugation at 4 °C for 15 min to sediment particulate materials. Protein concentrations were measured using a Bio-Rad protein assay kit (Bio-Rad Laboratories, Hercules, CA, USA). Proteins (30 μg) from whole cell extracts were separated on SDS-polyacrylamide gels and transferred onto nitrocellulose membranes. Membranes were blocked with 1% bovine serum albumin in TBS containing 0.05% Tween containing primary antibodies to Erk2, pErk, STAT3, pSTAT3, Akt, pAkt (Ser 473) caspase-3 and cleaved PARP and β-actin primary antibodies (Cell Signaling, Beverly, MA, USA). Blots were probed with antibodies to secondary antibodies conjugated with horseradish peroxidase (Cell Signaling) before visualization with enhanced chemiluminescence detection (Thermo Scientific, Rockport, IL, USA).

### 4.8. Immunohistochemistry

The liver tumor sections were fixed in 4% formalin and paraffin embedded. Immunohistochemistry staining for Vegf-A expression was carried out with polyclonal rabbit antibody (SC-152, Santa Cruz Biotech, Dallas, TX, US), and mouse monoclonal Vegf-R3 (Cat. No. 14-5988, ebioscience, San Diego, CA, USA), CD34 (14-0342, ebioscience), PCNA (SC-56). Sections were deparaffinized and rehydrated with series of xylene and alcohols followed by10 mM of citric acid antigen retrieval. For the monoclonal mouse antibodies mom block (MOM kit, BMK-2202, Vector labs, Burlingame, CA, USA) was used according to the manufacturer’s instructions. Sections were stained by appropriate biotinylated antibodies. Followed by Elite Vectastain ABC standard kit (PK6100, Vector labs) with an appropriate substrate nova red kit was used (SK 4800, Vector labs). Counterstain was performed with haemotoxylin.

### 4.9. In Vivo Tumor Model

MTD2 are transgenic mice that express SV40 T antigen (Tag) under the influence of major urinary promoter (MUP) leads to spontaneous, Tag expressing HCC throughout the liver [47]. Although MTD2 mice are genetically identical to C57BL/6 mice, due to central or peripheral tolerance, lack CD8+ T cell response to Tag, leading to tumor growth. Two groups of 8 mice each with tumors were included in the study. The maximum tolerated dose (MTD) of PBISe was demonstrated in previous experiments on F344rats, and Multiple Intestinal neoplasia (Min) mouse (Data not published). The MTD of PBISe in F344 rats when given in a diet was observed to be between 10 and 20 ppm (as Se), and MTD of PBISe when given by i.p. injection was 5 ppm (as Se), whereas the MTD of PBISe in Min mouse was between 2.5 ppm and 5 ppm (as Se). In our study we have used PBISe at a dose of 2.5 ppm (as Se). One group was given 2.5 ppm (as Se) conc. of PBISe per mice every alternate day for about six weeks. Second group was injected with Hanks Balanced Salt Solution (HBSS) that served as a control. MRI was used to demonstrate size of tumor with treatment every week for six weeks.

### 4.10. Statistical Analysis

All experiments were independently done in triplicate. The two-sample *t*-test was used to evaluate the effect change between the treatment and control groups at varying concentrations of the PBISe treatment. Multiple hypotheses testing correction is not provided due to the unquantifiable correlations present among the different hypotheses. A regression based statistical analysis was performed to quantify the effects of treatment, time, and their interaction on tumor size. The R program was used to perform the statistical analyses and generate the graphics (https://www.r-project.org). Data summaries are expressed as mean ± SE [48,49].

## 5. Conclusions

From the literature, selenium holds a great promise as a potential chemopreventive and chemotherapeutic agent for the treatment of human HCC. A further significant amount of effort is needed, before this selenium finds clinical usefulness in the treatment of HCC. Our studies on PBISe hold promising results on the effects on PI3 kinase, MAPK and STAT3 pathways, which are implicated in inflammation and hepatocarcinogenesis. However there is also a need to systematically demonstrate the chemopreventive and chemotherapeutic effects of selenium in human HCC in a clinical trial.
